# Consolations in Travel, or the Last Days of a Philosopher

**Published:** 1830-04-01

**Authors:** 


### CONSOLATIONS IN TRAVEL, OR THE LAST DAYS OF A PHILOSOPHER. By *Sir Humphry Davy*, Bart.

THE last days indeed of a philosopher! or rather the termination of a long course of chemical philosophy, in a short but splendid dream of metaphysical abstraction! The extent and versatility of a man's genius can never be known till they are tried. Who would have supposed that a writer to the Signet should be the writer of Marmion—or that a poet should be able to discard his mistresses, the Muses—nay even his fair "Lady of the Lake," and take to writing "Tales of my Landlord"? Sir Humphry Davy has exhibited still greater versatility of talent than Sir Walter Scott, or even Skakespeare. From analyzing salts and metals, he has rushed into the stars and planets, to describe the inhabitants of Jupiter and Saturn! From poring over the crucible in Albemarle Street, he takes a cruise on the back of a comet through unlimited space, descanting on the inhabitants, manners, and customs of the various orbs which he passes on his route, with as much ease as he would have delivered a lecture on the safety-lamp. It has been said of Shakespeare, that he—

"Exhausted worlds and then imagined new"—

but such was not the case. His Ariels and his Calibans were all of this world, whatever diversity of sentiment or action they displayed. But Sir Humphry Davy has far outstripped the bard of Avon, by making us intimately acquainted with beings of other worlds, and regions into which the human mind never before dared to pry. Whether the lamented philosopher will be hailed by the orthodox Christian or the sceptical free-thinker, as supporting one or other of their respective doctrines, we shall not attempt to determine; but we suspect that he is rather too Pythagorean for the divine, and too spiritual for the materialist.

Although the philosophy of other worlds is rather out of our beat; yet the last days and the last lucubrations of such a philosopher as Sir Humphry Davy, must excite some interest in the minds of even the most plodding practitioners in physic, and, therefore, we shall make no apology for introducing to our readers a short account of the very curious production now lying open before us.

It consists of six dialogues, the first being entitled the "VISION." The dialogue opens in the spacious arena of the Colosæum between two English friends, (and the author) the former a learned but liberal Catholic—the latter a free-thinking Aristocrat. It was not likely that the sentiments of these opposite characters should harmonize long, while remarking on the Pagan and Christian forms of religion. High blood was rising, when the author dismissed his friends, and remained to ruminate within the majestic ruins of Vespasian's amphitheatre. The following passage will make a favourable impression on the reader.

"It was a still and beautiful evening in the end of May; the last sun-beams were dying away in the western sky and the first moon-beams shining in the eastern; the bright orange tints lighted up the ruins, and as it were kindled the snows that still remained on the distant Appennines, which were visible from the highest accessible part of the amphitheatre. In this glow of colouring, the green of advanced spring softened the gray and yellow tints of the decaying stones, and as the lights gradually became fainter, the masses appeared grander and more gigantic; and when the twilight had entirely disappeared, the contrast of light and shade in the beams of the full moon and beneath a sky of the brightest sapphire, but so highly illuminated that only Jupiter and a few stars of the first magnitude were visible, gave a solemnity and magnificence to the scene which awakened the highest degree of that emotion which is so properly termed the sublime. The beauty and the permanency of the heavens and the principle of conservation belonging to the system of the universe, the works of the Eternal and Divine Architect, were finely opposed to the perishing and degraded works of man in his most active and powerful state. And at this moment so humble appeared to me the condition of the most exalted beings belonging to the earth, so feeble their combinations, so minute the point of space, and so limited the period of time in which they act, that I could hardly avoid comparing the generations of man, and the effects of his genius and power, to the swarms of luceoli or fire-flies which were dancing around me, and that appeared flitting and sparkling amidst the gloom and darkness of the ruins, but which were no longer visible when they rose above the horizon, their feeble light being lost and utterly obscured in the brightness of the moon beams in the heavens." 13.

Left to himself in the Colosæum, and aided by the light of the moon and the solitude of the scene, our author fell into a reverie on the awful vicissitudes that had befallen the once potent city of Rome, concluding that no new empire would ever again rise out of her colossal ruins—that the world itself was now, perhaps, in its zenith—and would soon be in decay. At this moment the philosoper fell into a kind of trance or dream—soft music sounded in his ears—a genius, invisible but audible, spoke in heavenly accents, and, after the usual preliminaries, and with the usual machinery, conducted the favoured dreamer through the air to a spot where a scene opened, and disclosed the original races of created beings. The surface of the earth was covered with forests and marshes—wild animals were grazing in extensive savannahs, while lions, tigers, and other ferocious beasts occasionally disturbed and preyed upon them—naked savages were feeding upon wild fruits, devouring shell-fish, and sometimes fighting with clubs for the remains of a whale. Their habitations were in caves, or under the shelter of the palm tree—and their greatest delicacies were the date and the cocoa-nut, for which they often contended in deadly strife. Some of the savages had weapons, pointed with flint or fish-bones, with which they destroyed other animals for food. The genius, of course, pointed the dreamer's attention to this scene, as that of "man in his newly-created state, full of youth and vigour." He was then hurried away through the air, when scene the second opened on his view. The earth was then partly cultivated and partly wild—men were covered with the skins of beasts—cattle were enclosed in pastures—corn was growing in the fields—cottages appeared in the vallies—and, in short, man was in the agricultural state, the golden age of the poets. Another darkness, and another whirl through the air, disclosed the third scene or act of the visionary drama. Then the philosopher saw extensive cultivated plains, large cities, palaces, forums, temples—groups of men performing military exercises—galleys ploughing the ocean—roads traversed by carriages—in short, a degree of civilization—but the instruments of brass—and poetry only *oration*. There was as yet, no written language.

In the next scene a great alteration was observable. Instruments of malleable iron were in use—and *thought* was rendered permanent by characters impressed on papyrus. Then it was that small bands of iron-clad men subdued thousands of savages—traversed the seas,—founded colonies—built cities—and carried with them, wherever they went, the arts of civilization. At this time, too, libraries were filled with rolls of papyrus—the same steel which destroyed armies in the field, struck out forms from the marble rock, "more perfect than those of life"—the walls of palaces and temples were covered with pictures portraying historical events, with the truth of Nature and the poetry of mind—in fact, man had arrived at that state of society which is still an object of admiration to the youth of modern times, and which has left maxims of war, policy, letters, and arts, which yet remain models for imitation.

"I opened my eyes, and recognized the very spot in which I was sitting when the vision commenced. I was on the top of an arcade under a silken canopy, looking down upon the tens of thousands of people, who were crowded in the seats of the Colosæum, ornamented with all the spoils that the wealth of a world can give; I saw in the arena below animals of the most extraordinary kind, and which have rarely been seen living in modern Europe, the giraffe, the zebra, the rhinoceros and the ostrich from the deserts of Africa beyond the Niger, the hippopotamus from the upper Nile, and the royal tiger and the gnu from the banks of the Ganges. Looking over Rome, which, in its majesty of palaces and temples, and in its colossal aqueducts bringing water even from the snows of the distant Appennines, seemed more like the creation of a supernatural power than the work of human hands; looking over Rome to the distant landscape, I saw the whole face as it were of the ancient world adorned with miniature images of this splendid metropolis. Where the Roman conquered, there he civilized; where he carried his arms, there he fixed likewise his household gods; and from the deserts of Arabia to the mountains of Caledonia there appeared but one people, having the same arts, language and letters, all of Grecian origin." 26.

The next view was, of course, Rome in ruins—pillaged by Goths and Vandals—her statues and temples demolished—her warriors sunk in effeminacy, and conquered by hordes of barbarians. Once more shifting the scene, he saw Italy recovering from her fallen state—learning stealing from the musty papers of the monks—and the press invented to render literature and science imperishable for the future. Gunpowder, too, had now changed the face of warfare, substituting skill and ingenuity for personal conflicts and brute force. It is needless to say that astronomy, chemistry, mathematics, machinery, and the wonderful effects of steam and gas, were pointed out by the genius—and that England was seen conspicuous in the van of intellect, art, and science. The following sentiments, from the mouth of the Genius, savour a little of the Pythagorean philosophy.

"In comparing the population of the globe as it now is with what it was centuries ago, you would find it considerably greater; and if the quantity of life is increased, the quantity of happiness, particularly that resulting from the exercise of intellectual power, is increased in a still higher ratio. Now, you will say, *is mind generated, is spiritual power created;* or, are those results dependent upon the organization of matter, upon new perfections given to the machinery upon which thought and motion depend? 'I proclaim to you,' said the Genius, raising his voice from its low and sweet tone to one of ineffable majesty, 'neither of these opinions is true. Listen, whilst I reveal to you the mysteries of spiritual natures, but I almost fear that with the mortal veil of your senses surrounding you, these mysteries can never be made perfectly intelligible to your mind. Spiritual natures are eternal and indivisible, but their modes of being are as infinitely varied as the forms of matter. They have no relation to space, and, in their transitions, no dependence upon time, so that they can pass from one part of the universe to another by laws entirely independent of their motion. The quantity, or the number of spiritual essences, like the quantity or number of the atoms of the material world, are always the same; but their arrangements, like those of the materials which they are destined to guide or govern, are infinitely diversified; they are, in fact, parts more or less inferior of the infinite mind, and in the planetary systems, to one of which this globe you inhabit belongs, are in a state of probation, continually aiming at, and generally rising to a higher state of existence. Were it permitted me to extend your vision to the fates of individual existences, I could show you the same spirit, which in the form of Socrates developed the foundations of moral and social virtue, in the Czar Peter possessed of supreme power and enjoying exalted felicity in improving a rude people. I could show you the monad or spirit, which with the organs of Newton displayed an intelligence almost above humanity, now in a higher and better state of planetary existence drinking intellectual light from a purer source and approaching nearer to the infinite and divine Mind." 44.

The philosopher now ascends through regions where we dare not follow him—except at a very respectful distance. He passed by the moon and stars so near that he could almost have touched them with his hand—and at length came to the verge of the immense atmosphere of Saturn.

"I saw below me a surface infinitely diversified, something like that of an immense glacier covered with large columnar masses, which appeared as if formed of glass, and from which were suspended rounded forms of various sizes, which, if they had not been transparent, I might have supposed to be fruit. From what appeared to me to be analogous to masses of bright blue ice, streams of the richest tint of rose-colour or purple burst forth and flowed into basins, forming lakes or seas of the same colour. Looking through the atmosphere towards the heavens I saw brilliant opaque clouds of an azure colour that reflected the light of the sun, which had to my eyes an entirely new aspect, and appeared smaller, as if seen through a dense blue mist. I saw moving on the surface below me immense masses, the forms of which I find it impossible to describe; they had systems for locomotion similar to those of the morse or sea-horse, but I saw with great surprise that they moved from place to place by six extremely thin membranes, which they used as wings. Their colours were varied and beautiful, but principally azure and rose-colour; I saw numerous convolutions of tubes, more analogous to the trunk of the elephant than to any thing else I can imagine, occupying what I supposed to be the upper parts of the body, and my feeling of astonishment almost became one of disgust, from the peculiar character of the organs of these singular beings; and it was with a species of terror that I saw one of them mounting upwards apparently flying towards those opaque clouds which I have before-mentioned." 48.

These elephantine beings turned out to be far superior to man. They had modes of perception of which we are ignorant—wider spheres of vision—more exquisite organs of touch—more refined pleasures—more intellectual pursuits. Their astronomical records went back a hundred times farther than ours. It was gratifying to find that, in Saturn, there were no wars—their only competitions being in pursuit of pure glory. The columnar masses, and even the opaque azure clouds, turned out to be works of art, connected with processes for the preparation of their food (for they resembled us a little in eating and drinking) and philosophical researches. We are unable to follow the philosopher through the cometary worlds; but collect that—"the universe is every where full of life, but the modes of this life are infinitely diversified, and yet every form of it must be enjoyed and known by every spiritual nature before the consummation of all things." Also that it is a divine law, that although our spiritual natures rise through different stages of planetary life, they leave their dust behind them, and only carry with them their intellectual powers.

"You ask me if they have any knowledge or reminiscence of their transitions; tell me of your own recollections in the womb of your mother and I will answer you. It is the law of divine wisdom that no spirit carries with it into another state and being any habit or mental qualities except those which may be connected with its new wants or enjoyments; and knowledge relating to the earth would be no more useful to these glorified beings than their earthly system of organized dust, which would be instantly resolved into its ultimate atoms at such a temperature." 56.

Finally, he was informed by the genius that the love of knowledge or of intellectual power is, in its final development, the love of God—and that the future destinies of our souls depend upon the manner in which this love of knowledge is exercised in this state of existence. When well applied we mount higher in the planetary systems—when the reverse, we "sink in the scale of existence," and continue on earth, or in some inferior planet, "till our errors are corrected by painful discipline."

It is true that in the succeeding dialogue, these doctrines of the genius are combated by Ambrosio, his Catholic companion, and the philosopher appears to be converted by the arguments of his opponent; but the impression left on the mind of the reader is, that the scheme sketched out by the genius is that to which the philosopher had brought his mind by long reflexion, while the arguments of Ambrosio appear inadequate to so sudden a conversion of the author.

It is not a little curious that a man who has been all his life experimenting upon *matter*, and consequently pursuing researches the opposite to those of metaphysics—especially these ultra-metaphysics relating to the future state of the soul and the beings of other orbs, should all at once ascend from this lowly earth, and range excursive through the regions of Fancy. In the third dialogue, the subject of geology, and the history of the Earth are taken up, and some ingenious remarks and speculations thrown out. As these topics are more nearly allied to medicine than those just reviewed, we shall dedicate a short article to the remainder of Sir Humphry's work in our next number.

---

